# Restoring vision after cat bite: a case report on successful diagnostic and therapeutic regimen for *Capnocytophaga* endophthalmitis

**DOI:** 10.1186/s12348-023-00378-7

**Published:** 2024-01-22

**Authors:** Lasse Wolfram, David A. Merle, Jonas Neubauer, Spyridon Dimopoulos

**Affiliations:** 1 Department for Ophthalmology, University Eye Clinic, Eberhard Karls University of Tübingen, Elfriede-Aulhorn-Straße 7, 72076 Tübingen, Germany; 2 Department for Ophthalmology, Institute for Ophthalmic Research, Eberhard Karls University of Tübingen, Elfriede-Aulhorn-Straße 7, 72076 Tübingen, Germany; 3 Eye Center, Medical Center, Faculty of Medicine, University of Freiburg, Killianstraße 5, Freiburg, 79106 Germany; 4 Department of Ophthalmology, Medical University of Graz, Auenbruggerplatz 2, 8036 Graz, Austria

**Keywords:** Case report, *Capnocytophaga*, Endophthalmitis, Pars plana vitrectomy, Cataract surgery

## Abstract

**Background:**

*Capnocytophaga* is a bacterium frequently found in the oral flora of dogs and cats (e.g. *Capnocytophaga canimorsus*) and humans (e.g. *Capnocytophaga gingivalis*). Among *Capnocytophaga* related ocular infections, fulminant endophthalmitis is a rare but sight-threatening clinical manifestation.

**Case presentation:**

A 35-year-old previously healthy patient presented after a cat bite into the left upper and lower eyelid and nasal part of the conjunctiva of the left eye. At initial consultation, the corrected visual acuity was 0.8 in decimal scale and a detailed clinical examination revealed no evidence of ocular penetration. However, daily follow-up examinations under local therapy revealed a progressive intraocular inflammation, therefore the decision was made to perform a diagnostic vitrectomy with intravitreal and systemic antibiotic treatment. *Capnocytophaga felis* was detected as the cause of endophthalmitis and the initiated treatment resulted in quick morphological and functional recovery of the left eye. After surgery of secondary cataract, visual acuity improved from hand motion preoperatively to 1.0 postoperatively.

**Conclusions:**

Early recognition as well as prompt and effective treatment of animal bite associated endophthalmitis is essential for good visual recovery and functional outcome. Furthermore, this case highlights the importance of daily follow-up examinations, even in the absence of signs of ocular penetration and intraocular inflammation, to enable prompt and effective treatment initiation. Given the negative results in bacterial culture, we additionally emphasize the value of sequencing-based microbiological diagnostics in unclear cases.

## Background

*Capnocytophaga spp.* are capnophilic, microaerophilic, gram-negative bacilli, frequently found in the oral flora of dogs and cats (e.g. *Capnocytophaga canimorsus* [1, 2]) and humans (e.g. *Capnocytophaga gingivalis* [3, 4]). Predominantly in the course of bites, scratches, licks or mere exposure to these animals, *Capnocytophaga* can lead to various types of local and systemic infections, including lethal sepsis [5, 6]. First cases of endophthalmitis caused by *Capnocytophaga* were published in 1993 and only a limited cumulative number of eight cases of *Capnocytophaga* related endophthalmitis have been described so far [7–12]. Here we present a clinical case of a successful diagnostic and therapeutic approach to *Capnocytophaga* endophthalmitis after a cat bite.

## Case presentation

**Fig. 1:**
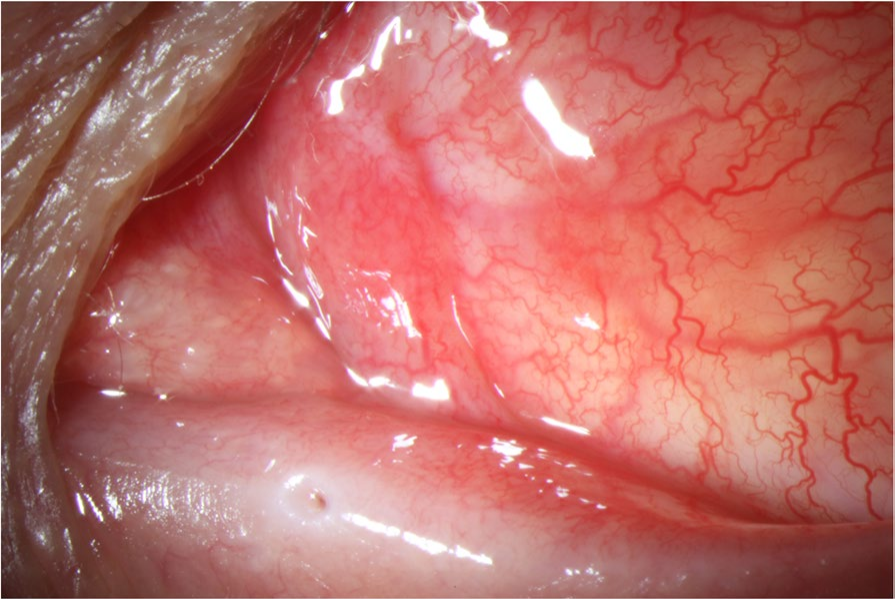
Conjunctival tear of approximately 3 mm in length with conjunctival injection and chemosis at initial consultation (slit lamp examination)

**Fig. 2:**
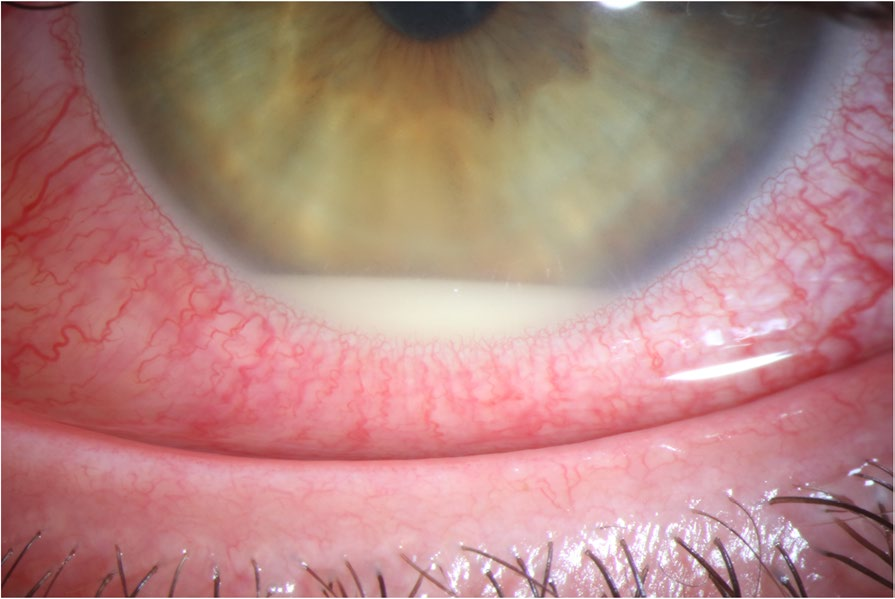
Anterior chamber inflammation with a hypopyon on the second day after injury (slit lamp examination)

**Fig. 3:**
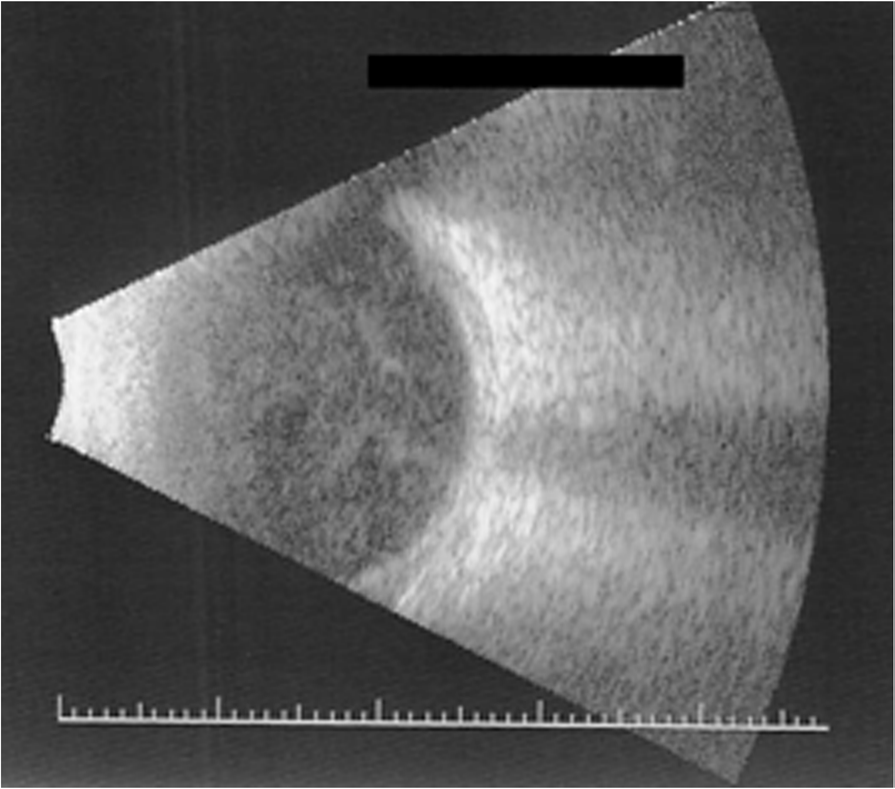
Infiltration of the vitreous on the second day after injury (ocular ultrasound)

**Fig. 4:**
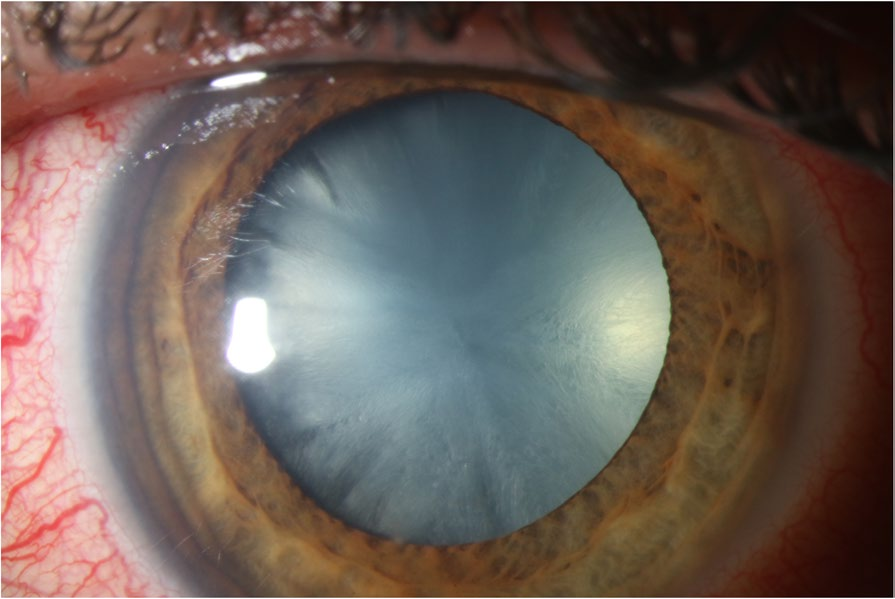
Secondary cataract three weeks after injury (slit lamp examination)

**Fig. 5:**
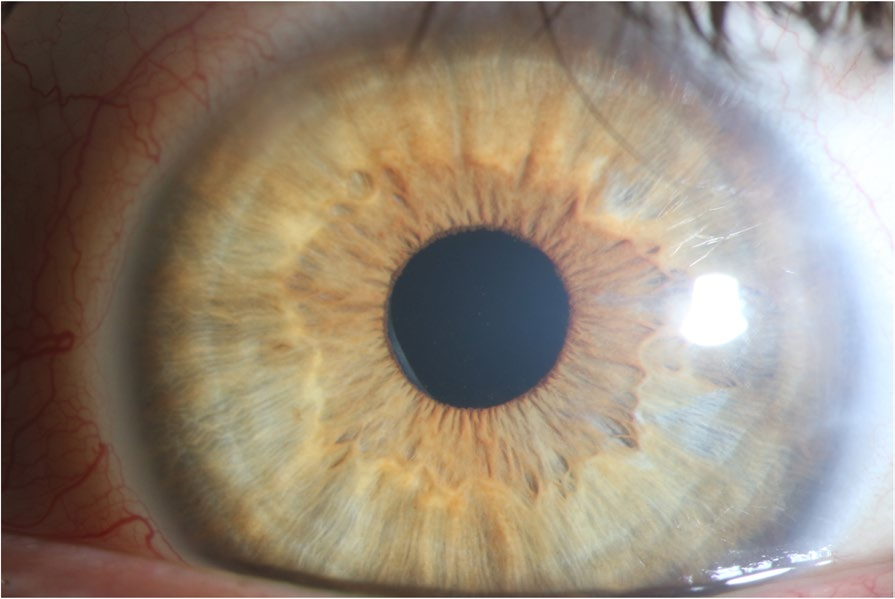
Result after cataract surgery with implantation of the intraocular lens into the sulcus (slit lamp examination)

A 35-year-old previously healthy patient presented after a cat bite into the left upper and lower eyelid and nasal part of the conjunctiva of the left eye. At initial consultation, the corrected visual acuity was 0.8 in decimal scale. Clinical examination revealed scratches of the lids and a conjunctival tear of approximately 3 mm in length with conjunctival injection and chemosis (Fig. 1). Although a sclera wound after a cat bite is not always easy to identify, we decided to perform a sclera exploration in operating theater. During this procedure, no signs of penetration could be seen. The patient described that he had got bitten into his left eye during play with his own cat. He had been vaccinated against tetanus and his cat had got all regular vaccinations. Since initially no intraocular inflammation was seen, the affected eye was treated with moxifloxacin eye drops (5 mg/ml) and the eyelids with gentamicin eye ointment (5 mg/g), each applied five times a day. The following day, mild inflammation of the anterior chamber was detected. Therefore, dexamethasone (1 mg/ml) four times a day was added to the local antibiotic therapy. Despite local treatment, the intraocular inflammation increased rapidly, showing anterior chamber inflammation with a hypopyon (Fig. 2) and vitreous infiltration (Fig. 3) on the second day after injury. We admitted the patient and initiated intravenous wide spectrum antibiotic treatment with ceftazidime 1 g twice a day and imipenem/cilastatin 1 g three times a day. At that point, visual acuity was reduced to perception of hand motion only. To avoid posterior synechiae formation, we added cyclopentolate 10 mg/ml twice a day to the local therapy. On the fourth day after injury, a diagnostic pars plana vitrectomy was performed in order to obtain samples for microbiological analysis. During vitrectomy, vancomycin (1 mg/0.1 ml) and ceftazidime (2.25 mg/0.1 ml) were applied intravitreally. The patient recovered quickly after surgery and empiric antibiotic treatment. The vitreous punctate was subjected to culture analysis for aerobic, anaerobic and fungal pathogens, yielding negative results in the absence of antimicrobial substances. Utilizing the Sanger sequencing method for 16S rDNA and species identification through both NCBI (non-curated database) and EzBioCloud (curated database), we identified *Capnocytophaga felis* as the cause of endophthalmitis. We changed the therapeutic regimen accordingly from intravenous to oral treatment with clindamycin 600 mg three times a day and discharged the patient five days after surgery. A secondary cataract was identified three weeks after the injury (Fig. 4). Since during the vitrectomy, no signs of any lens touch or penetration could be seen, we strongly believe that the rapid cataract development was associated with the primary injury. A cataract surgery was performed. During surgery, a penetration of the posterior capsule was observed, confirming our hypothesis of the injury having caused the cataract development. An implantation of the intraocular lens into the sulcus was therefore necessary (Fig. 5). Already one day after cataract surgery, visual acuity improved to 1.0 in decimal scale and remained stable during follow up examinations.

## Discussion and conclusions

In the presented case, early recognition along with prompt and effective treatment of the intraocular *Capnocytophaga* infection were crucial to preserve visual outcome. Even though no signs of ocular penetration were visible in the first place, clinical aspects, e.g. massive intravitreal inflammation despite intensive local antibiotic treatment and the rapid development of a secondary cataract after the trauma with a capsular defect diagnosed later, strongly suggested an eye penetration during cat bite injury. Initial daily follow-up examinations allowed us to respond quickly to the developing endophthalmitis, which otherwise would have potentially led to irreversible visual loss. Therefore, we recommend a strict control regimen after bite injuries, even if no obvious signs of ocular penetration can be detected.

Due to its rapid and aggressive progression, infectious endophthalmitis is a severe vision threatening disease [13]. In order to avoid retinal toxicity, an initial empiric treatment with local broad-spectrum antibiotic, like moxifloxacin, as well as intravitreal and intravenous treatments with broad-spectrum antibiotics can be necessary. Due to a progressive intraocular inflammation, we treated the patient with intravenous ceftazidime and imipenem/cilastatin. This combination has high potency especially in gram-negative endophthalmitis, including *Capnocytophaga* related endophthalmitis [7, 14, 15]. The combination of intravitreally applied vancomycin and ceftazidime covers a broad gram-positive and gram-negative spectrum and is known to be an ideal empiric treatment of endophthalmitis [15], under which the patient was already recovering well. As comprehensive culture analysis of vitreous punctate for aerobic, anaerobic and fungal pathogens did not show any growth, sequencing-based diagnostic was crucial in this case to detect *Capnocytophaga felis*, a recently newly described species isolated from the oral cavity of cats [16], as the cause of endophthalmitis. This was especially important to optimize antibiotic regimen to oral treatment with clindamycin, as recommended for the treatment of *Capnocytophaga* related ocular infections [9, 17] in order to prevent severe systemic infections, e.g. *Capnocytophaga* associated lethal sepsis [6]. Taken together, early recognition as well as prompt and effective diagnosis and treatment of the developing endophthalmitis were key to achieve best therapeutic outcome.

## Data Availability

The datasets used and analyzed during the current study are available from the corresponding author on reasonable request.
